# Cost-effectiveness analysis: nonsurgical root canal treatment versus single-tooth implant

**DOI:** 10.1186/s12903-023-03173-x

**Published:** 2023-07-15

**Authors:** Hai-Ling Zang, Yu Zhang, Xiao-Wen Hao, Li Yang, Yu-Hong Liang

**Affiliations:** 1 Department of Cariology and Endodontology, Peking University School and Hospital of Stomatology & National Engineering Research Center of Oral Biomaterials and Digital Medical Devices, Beijing, China; 2 Department of Stomatology, Peking University International Hospital, Beijing, China; 3 Department of Implantology, Peking University School and Hospital of Stomatology & National Engineering Research Center of Oral Biomaterials and Digital Medical Devices, Beijing, China; 4 Department of Health Policy and Management, School of Public Health, Peking University, NO.38 Xueyuan Road, Haidian District, Beijing, 100191 China

**Keywords:** Cost-effectiveness, Single-tooth implant, Nonsurgical root canal treatment, Propensity score matching

## Abstract

**Background:**

Economic evaluation of nonsurgical root canal treatment (NSRCT) and single-tooth implant (STI) provides useful information for medical decision. This retrospective study aimed to evaluate the cost-effectiveness of NSRCT versus single-tooth implant (STI) after 5-year treatment in a university affiliated hospital in Beijing, China.

**Methods:**

211 patients who underwent NSRCT and 142 patients who had STI were included and recalled after 5-year treatment. The propensity scores were used to match the cases of two treatment modalities. At recall, outcomes were determined based on clinical and radiographical examinations. For endodontically treated cases, absence or reduction of radiolucency were defined as success. Marginal bone loss (MBL) ≤ 4 mm were determined as success for implant cases. Direct and indirect costs were calculated in China Yuan (CNY). Patients’ willingness to pay (WTP) for each treatment modality was evaluated by questionnaires. A cost-effectiveness analysis was performed from the societal perspective.

**Results:**

170 patients with 120 NSRCT teeth and 96 STI were available at recall. Based on propensity score matching, 76 endodontically treated teeth were matched to 76 implants. Absence of the radiolucency was observed in 58 of 76 endodontically treated teeth (76%) and reduction of the radiolucency in 9 of 76 teeth (12%) and altogether the success rate was 88%. 100% implants were detected with marginal bone loss (MBL) ≤ 4 mm. The cost advantage of NSRCT (4,751 CNY) over STI (20,298 CNY) was more pronounced. Incremental cost effectiveness ratio (ICER) was 129,563 CNY (STI-NSRCT) per success rate gained. It exceeded the patients’ willingness to pay value 7,533 CNY.

**Conclusions:**

Clinical outcomes of NSRCT and STI could be predictable after 5-year treatment. NSRCT may be more cost-effective than STI for managing endodontically diseased teeth.

## Background

Pulp and periapical diseases have an impact on oral health and quality of life of individuals [1]. Nonsurgical root canal treatment  (NSRCT) is the most common used treatment modality which aims to resolute periapical pathoses, retain the natural tooth and preserve the natural dentition [2–5]. Over the past couple of decades, considerable advances have been made in the theory and technology of single-tooth implant treatments (STI), which has also become an alternative intervention for teeth with pulpal or periapical pathology. By removing the questionable tooth and replacing it with an implant, this surgical approach can meet the prosthetic and esthetic demands of patients [6, 7]. To restore a compromised tooth with NSRCT or replace it with STI is a frequent and challenging dilemma that clinicians and patients encounter in dental practice [8–13]. The decision-making process is mostly influenced by operator’s clinical experience, short-term ‘success rate’, patient demographic attributes and immediate cost of each treatment modality [3, 14–17]. Moreover, health policymakers, governments and socioeconomic development also have an effect on the decision-making process to some extent [18].

As a supplement of the traditional criteria of the outcome of medical interventions, cost-effectiveness analysis aims to quantify the cost and effectiveness of medical technologies [19, 20]. Faced with insufficient medical resources, limited insurance and great medical service demands, it’s helpful to provide evidence for making reasonable decisions, prioritizing services and achieving reasonable allocation of limited medical resources.

What patients are most concerned about is the long-term cost and effectiveness, whereas there was few research focusing on the cost-effectiveness of NSRCT and STI [14]. Moreover, differences exist in culture, economic level and medical background in different countries, the economic information of NSRCT and STI in China is lacking. Combination of clinical outcome and economic evaluation is helpful for the healthy development of the medical and health industry. Therefore, the aim of this retrospective cohort study was to compare the cost effectiveness of NSRCT and STI for endodontic diseases.

## Methods

### Patient inclusion

This retrospective cohort study protocol was approved by the ethics board of Peking University Hospital of Stomatology, Beijing, China (no. PKUSSIRB-201951172). Patients treated between June 2014 to December 2015 in the Department of Cariology and Endodontics or Implantology of the hospital were included according to the following criteria. Patients were eligible for the study if they were at least 18 years of age with a complete demographic and medical history. All teeth were diagnosed as pulp and periapical diseases (e.g. acute/chronic pulpitis/ apical periodontitis, teeth with residual roots or crowns) and had received nonsurgical root canal treatment or single-tooth implant surgery and subsequent single-tooth restoration. Each endodontically treated tooth or implant had to have at least one adjacent natural tooth. Pregnant women, patients with poorly controlled systemic diseases, multi-unit restorations were excluded. Written informed consent was obtained from all patients at the time of enrollment.

### Clinical and radiographic evaluation

The patients were recalled after 5 years treatment through multiple telephone calls. At the recall examination, pain, swelling, sinus tract formation, tenderness to apical, gingival palpation or percussion, mobility as well as periodontal pocket formation were recorded. Periapical radiographs (PA) were obtained with the digital imaging system Digora Optime (Soredex, Helsinki, Finland) using a parallel technique.

Outcome was assessed based on clinical and radiographic measures. A periapical lesion was determined when disruption of the lamina dura was detected and the radiolucency associated with the radiographic apex was at least twice the width of the periodontal ligament space [21]. Two endodontists were trained and independently examined the PA images of endodontically treated teeth twice in 10% radiographs. In cases of disagreement, discussion was made until a consensus was achieved. The radiographic outcome is presented in 6 categories: no lesions pre and post treatment, new lesions, absence, reduction, enlargement, or unchanged [22]. Endodontically treated teeth were defined as success if there was no clinical signs and radiographic evidence showed absent/resolved or reduced periapical radiolucency. The Cohen kappa was used to assess inter-examiner and intra-examiner agreement.

An independent examiner, not related to the patients’ treatment, analyzed all radiographs, baseline and 5 years postoperatively, to measure the marginal bone loss (MBL) of the implant using Sketchpad software. The implant–abutment junction plane was chosen as a fixed reference level. The distance between this reference point and the marginal bone-to-implant level was measured in millimeters at the mesial and distal site. To correct for distortion errors due to the positioning of the radiograph in the mouth of the patient, all measurements were calibrated according to the actual length of the implant. The marginal bone levels were evaluated by comparing the bone levels measured at the time of prostheses placement with those measured during the follow-up periods. The marginal bone loss (MBL) was determined using the average value of the mesial and distal radiologic bone level. 10% radiographs were randomly selected and measured twofold to analyze intra-examiner agreement. The radiographic outcome is presented in 4 categories: MBL < 2 mm; 2 mm ≤ MBL ≤ 4 mm; MBL > 4 mm; MBL > 1/2 implant length [23]. For implants, MBL ≤ 4 mm, including MBL < 2 mm or 2 mm ≤ MBL ≤ 4 mm without symptoms were defined as success. The intraclass correlation coefficient (ICC) were used to assess intra-examiner agreement.

### Willingness to pay (WTP)

A questionnaire measured the maximum amount of money the patients would be willing to pay for the dental care (nonsurgical root canal treatment and implant) per year with a starting cost basis of ¥200, increments were added until more than ¥5,000. The answer options were: ‘200 CNY’, ‘500 CNY’, ‘1,000 CNY’, ‘2,000 CNY’, ‘5,000 CNY’, and ‘more than 5,000 CNY’. The average mean value was recorded as WTP per year and five times of the value was recorded as WTP for five years.

### Propensity score matching (PSM)

Nearest neighbor 1:1 propensity score matching for the following 4 variables was performed for cases of NSRCT and those of STI: age, sex, tooth type, jaw.

### Health outcomes and estimation of costs

The measure of effectiveness was the 5-year outcome (success rate) of NSRCT and STI. From a societal perspective, the costs of two treatment modalities were calculated, including the direct and indirect costs per tooth/implant. The costs were calculated in Chinese Yuan (CNY). Direct dental health care costs were comprised of registration, imaging, endodontic, surgical and prosthodontic procedure, prescription fees occurred at any time before and after the final restoration of NSRCT and STI. For cases requiring subsequent treatment intervention, including endodontic, adjunctive surgical and prosthetic complications, the costs were recorded. The transportation expenses were negligible and ignored in this study. Time costs were recorded for dental care in scheduled or unscheduled visits during 5 years. The patient’s time spent for treatment was assumed to be 0.5 day per visit. Beijing average wage in society of each year from 2014 to 2020 was applied for costs of patients’ time in treatment. The discount rate was 5%. The costs occurred any time before the final restoration were recorded as initial cost. The costs for complication intervention and follow-up after final restoration were recorded as maintenance costs.

### Cost-effectiveness analysis

The matched cases of each treatment modality were used for cost-effectiveness analysis. Incremental cost-effectiveness ratio (ICER) is used to express cost differences per gained effectiveness when comparing the two medical strategies. ICER was calculated using the formula.

ICER=∆c/∆e,

with ∆c means difference value of cost, ∆e means difference value of effectiveness. ICER of STI versus NSRCT was calculated and it was determined as the ratio of difference value of the fee for that individual treatment divided by the difference value of success probability of each individual treatment modality. Sensitivity analysis was performed to allow for the possible variation in each treatment modality, taking the variety of cost estimates into consideration. The 20% change of costs of different treatment modalities was used for this purpose.

## Results

170 patients (216 teeth) were available for at 5-year review. The demographic information of participants associated with NSRCT and STI is presented in Table 1. An assessment of the periapical radiography revealed that the inter-examiner kappa value was 0.942, and the intra-examiner kappa values were 0.846 and 0.815, respectively. The intra-examiner ICC values for the MBL measurements were 0.924.

**Table 1:** Information of Included and Reviewed Cases Associated with NSRCT and STI

Modalities	NSRCT	STI	Total
**Included patient/tooth**	211/247	142/155	353/402
**Recalled patient/tooth**	87/120	83/96	170/216
**Recall rate patient/tooth**	41%/49%	58%/62%	48%/54%
**Mean recall time (y)**	5.6	5.3	5.5
**Female/male**	57/30	52/31	109/61
**Median age (range) (y)**	38(19–72)	47(23–68)	43(19–72)

NSRCT: nonsurgical root canal treatment; STI: single-tooth implant

**Fig. 1:**
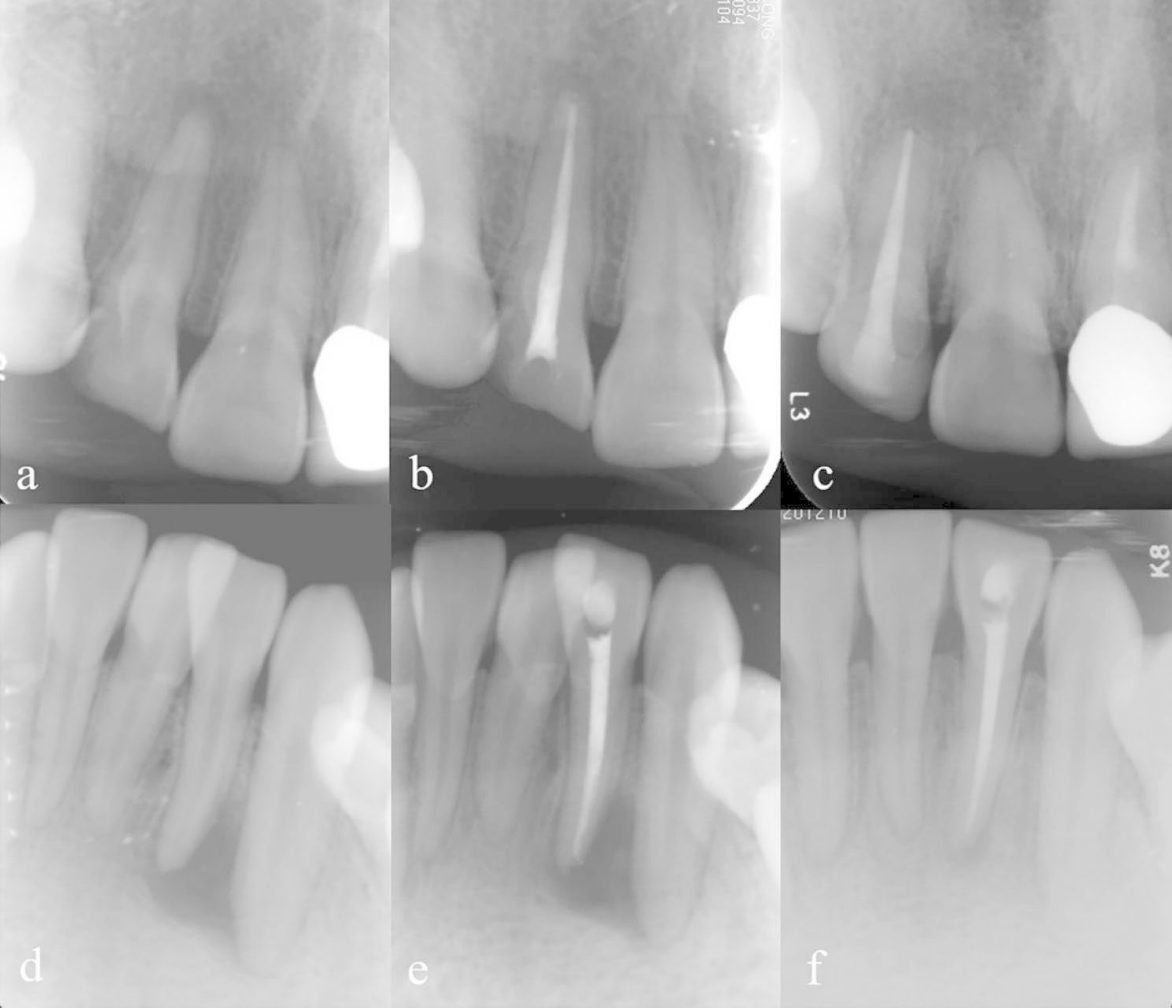
Representative successful cases of nonsurgical root canal treatment (NSRCT). (a-c) A 36-year-old female with a periapical lesion on right maxillary lateral incisor. (**a**) received root canal treatment and resin restoration (**b**). A radiograph of tooth 12 showed a flush root filling and no void was observed. There was no clinical signs or symptoms at 5 years recall after treatment. The postoperative PA images revealed resolved periapical lesions (**c**) and the case was determined as success. (**d-f**) The preoperative (**d**) and postoperative (**e**) PA images of tooth 32, which was diagnosed as apical periodontitis and underwent root canal treatment and received resin restoration. The periapical radiolucency reduced at the 5-year follow-up evaluation (**f**) and the case was determined as success

**Fig. 2:**
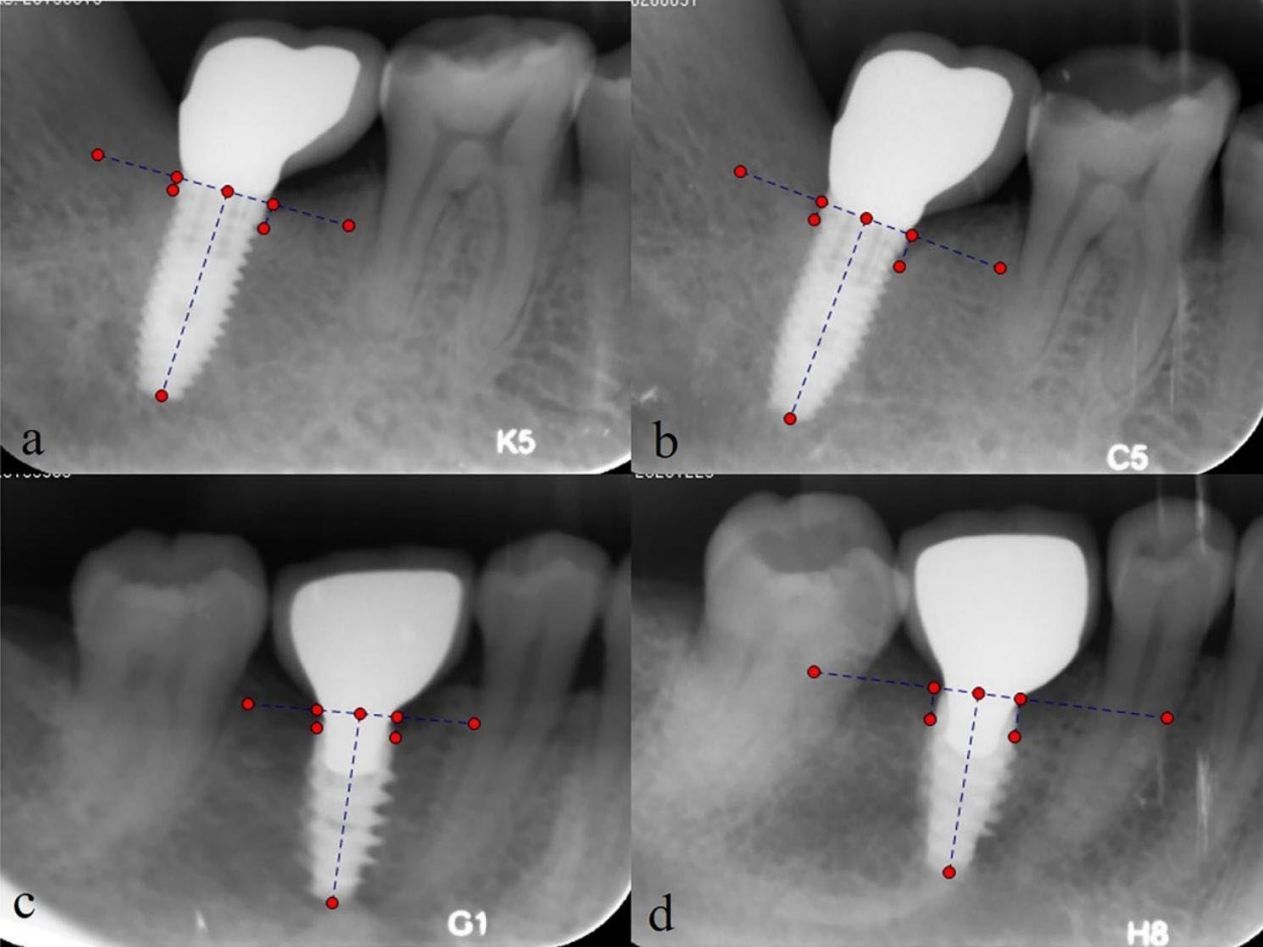
Representative successful cases of single-tooth implant (STI). (**a-b**) A 49-year male with tooth 47 received implant treatment. Radiographic illustrations of a case with marginal bone loss of 1.0 mm. Baseline (crown placement) (**a**) and 5 years of follow-up (**b**). (**c-d**) A 41-year female with tooth 46 received implant treatment. Radiographic illustrations of a case with marginal bone loss of 2.1 mm. Baseline (crown placement) (**c**) and 5 years of follow-up (**d**)

After 1:1 propensity score matching, 152 cases were matched. None of the variables such as sex, tooth type, jaw, age demonstrated significant differences between NSRCT and STI cases (P > .05) (Table 2). In endodontically treated teeth, 75 of 76 (99%) were symptom free. The absence of the radiolucency was observed in 58 teeth (76%) and reduction of the radiolucency in 9 teeth (12%) (Fig. 1). Altogether the success rate was 88%. The remaining one tooth which received NSRCT was symptomatic with swelling and showed enlarged lesion radiographically. All 76 implants survived without symptoms and showed marginal bone loss (MBL) ≤ 4 mm. 72 implants (95%) of them had a bone loss of < 2 mm (Fig. 2).

**Table 2:** Baseline Characteristics and Distribution of Cases (NSRCT and STI) before and after Propensity Score Matching

		NSRCT	STI	P
**Before matching**	**No. of teeth/implants**	120	96	
	**Age(y)**	41.41 ± 12.73	46.84 ± 9.577	< 0.001
	**Sex**			
	Male	42(35%)	36(37.5%)	0.704
	Female	78(65%)	60(62.5%)	
	**Tooth type**			
	**Anterior**	22(18.3%)	11(11.5%)	0.023
	**Premolar**	35(29.2%)	17(17.7%)	
	**Molar**	63(52.5)	68(70.8%)	
	**Jaw**			
	Maxillary	64(53.3%)	39(40.6%)	0.063
	Mandibular	56(46.7%)	57(59.4%)	
**After Matching**	**No. of teeth/implants**	76	76	
	**Age(y)**	46.57 ± 11.20	45.16 ± 9.51	0.761
	**Sex**			
	Male	28(36.8%)	32(42.1%)	0.507
	Female	48(63.2%)	44(57.9%)	
	**Tooth type**			
	Anterior	8(10.5%)	11(14.5%)	0.110
	Premolar	24(31.6%)	13(17.1%)	
	Molar	44(57.9%)	52(68.4%)	
	**Jaw**			
	Maxillary	35(46.1%)	34(44.7%)	0.871
	Mandibular	41(53/9%)	42(55.3%)	

Nonsurgical Root Canal Treatment; STI: Single-Tooth Implant

**Fig. 3:**
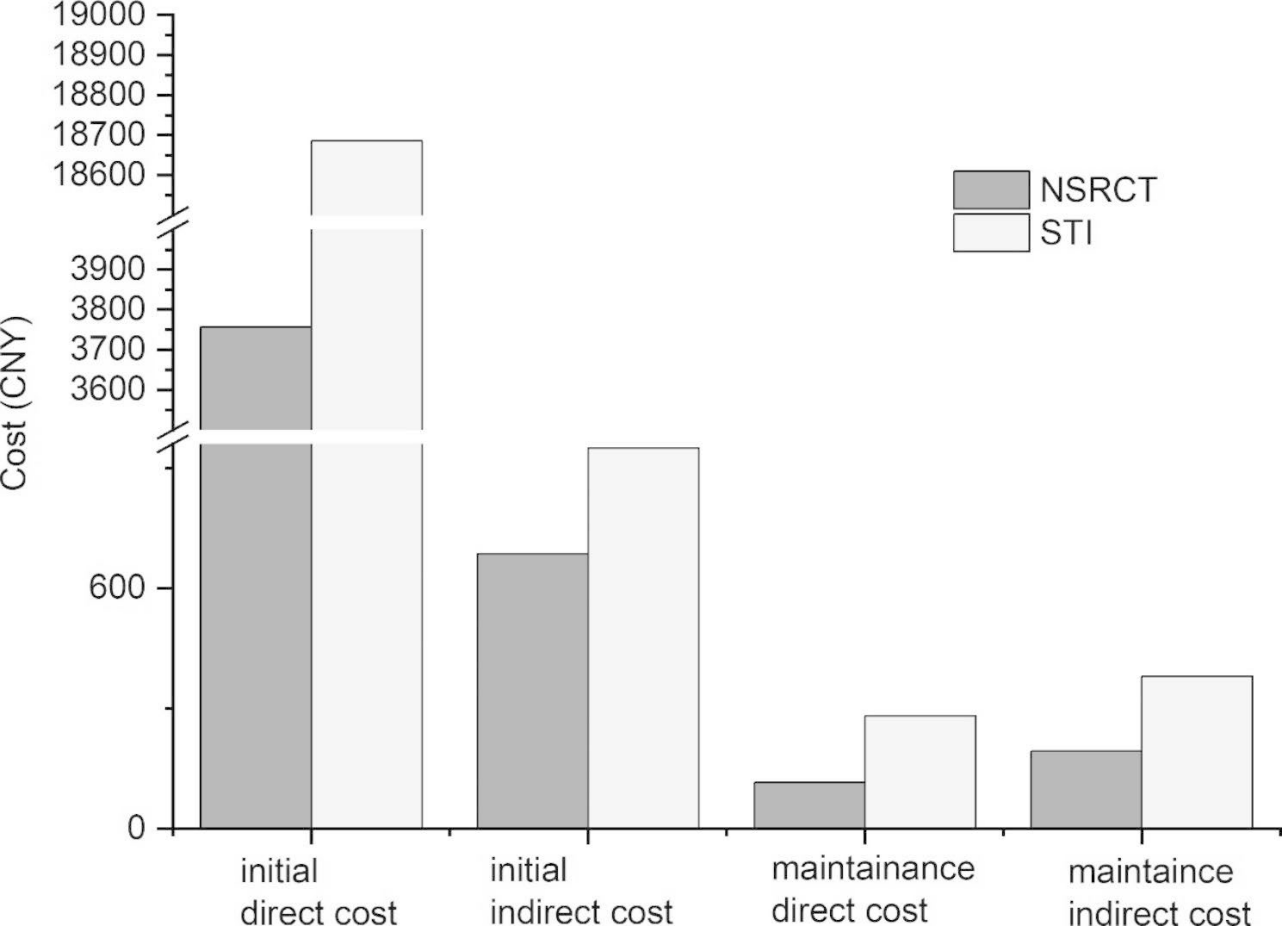
Mean (SD) Costs of NSRCT or STI per Tooth

Mean total costs per endodontically treated tooth/single-tooth implant were 4,751 and 20,298 CNY, respectively. The direct costs of NSRCT and STI account for 94% and 97% of their initial costs. Maintenance costs of NSRCT were 308 CNY, lower than that of STI 662 CNY (Fig. 3). The willingness to pay (WTP) mean value for 5 years was 7,533 CNY.

The incremental cost-effectiveness ratio (ICER) for STI vs. NSRCT was 129,563 CNY per success rate gained, which exceeded the patients’ willingness to pay value 7533 CNY. NSRCT was more cost-effective than STI. The sensitivity analysis revealed that taking the 20% change of costs of different treatment modalities into account, the ICER change range was 68,590 ~ 95,232 CNY, which still far exceeded the patient’s willingness to pay. NSRCT was still more cost-effective than STI. Initial direct cost of NSRCT and STI had a low impact on ICER.

## Discussion

To our knowledge, this is the first study to evaluate the cost-effectiveness of dental implants to a matched group of endodontic restorations in Peking University School and Hospital of Stomatology. Age, sex, jaw and tooth type were used for propensity score matching, which aimed to control confounders and minimize selection bias inherent to the nonrandomized and retrospective study design for two treatment modalities.

The credibility of the cost-effectiveness analysis depends on the credibility of the data quality and sources, including cost and effectiveness. In the present study, effectiveness data is the outcome of teeth or implants from a real-world clinical trial. An effectiveness criterion is defined as a dichotomic endpoint: success/failure. Clinical and radiographic examination were conducted to evaluate the outcome of endodontic and implant treatment. The 6-grade and 4-grade standards were used for NSRCT and STI, respectively [22, 23]. The grading standard could facilitate the analysis of different clinical endpoints and reduce the pressure of evaluators.

All costs associated with the dental treatments of the recalled patients’ teeth/implants during 5-year follow-up time were calculated, including direct and indirect medical costs. The current study establishes that total mean cost of the NSRCT is 4,751 ± 2,617 CNYs per patient (medium: 4,447; P25%~75%: 2,452 ~ 6,589). In this retrospective study, resin restoration, onlay and crown were placed to restore the endodontically treated teeth. And the cost showed wide variability. Of the 76 teeth included, 47 (61.8%) teeth had been restored by permanent restorations such as full crowns, onlays and post-core crowns, with an average initial direct medical cost of 5,152 CNY. While the other 29 (38.2%) teeth were restored by resin or temporary sealing materials, with an average value of 1,767 CNY. With more visits for permanent restorations, the indirect cost of 796 CNY is higher than the cases restored by resin of 483 CNY and the temporary sealing material of 344 CNY.

For implant cases, total mean cost is 20,298 ± 4,331 CNYs per patient over 5 years (medium: 18,910; P25%~75%: 17,501 ~ 22,333). The initial direct medical cost of 48 teeth without auxiliary surgery was 16,667 CNY and 28 teeth with auxiliary surgery was 22,147 CNY. It should be noted that the proportion of implant requiring post-intervention was 10.5% (8/76), higher than NSRCT, which was only 2.6% (2/76). And the result is similar to Hannan’s study, which was 12.4% for implants and 1.3% for NSRCT teeth, respectively [23]. Moreover, the treatment cost of implant complications, such as re-manufacturing of crowns and surgery of peri-implantitis, was also expensive, which ranges from 2,155 to 8,095 CNY. As a result, the maintenance cost of STI was higher than NSRCT.

Cost-effectiveness analysis was conducted to provide patients, clinicians, insurers, governments and other policymakers with economic information to help prioritize services and allocate resources. The incremental cost-effectiveness ratio (ICER) is the ratio of incremental cost to incremental effectiveness, expressing the cost difference per gained or lost effectiveness. In the present study, with different success criteria used, ICER of STI versus NSRCT was 81,829 CNY and 129,563 CNY, respectively. The detailed calculation methods were as follows: In the formula, ICER=∆c/∆e, ∆c = cost (STI) – cost (NSRCT) = 20,298.1- 4,750.5 = 15,547.6 CNY. When absence of radiolucency and MBL < 2 mm were determined as success, ∆e = effectiveness (STI) – effectiveness (NSRCT) = 95% − 76%=19%, ICER = 15,547.6 / 19%=81,829 CNY; When absence/reduction of radiolucency and MBL ≤ 4 mm were defined as success, ∆e = effectiveness (STI) – effectiveness (NSRCT) = 100% − 88%=12%, ICER = 15,547.6 / 12%=129,563 CNY. That means, for every successful treatment of one more tooth, the additional cost was 81,829 CNY and 129,563 CNY. These values exceeded the patient’s willingness to pay 7,544 CNY, which means NSRCT was more cost-effective than STI. In the current study, the sensitivity analysis was performed using the value within the quartile range of the current cost and NSRCT was still more cost-effective than STI, which verified the robustness of the result.

The costs incorporated within the current study were specific to the healthcare system currently operating in Beijing, China. The cost of NSRCT and STI was significantly different, whereas the difference of outcome between the two treatment modalities were little. When the cost gap between NSRCT and STI were reduced against different medical backgrounds, the value of ICER would also change accordingly. For instance, in a study conducted in the USA, each patient spent more than twice as much ($2,649.61 vs. $1,176.12) to have a functional STI compared with NSRCT [24]. This includes the fees for all adjunct and additional procedures as well as prescribed medications. To date, there have been four studies comparing the endodontic treatment outcomes and single-tooth implant and the result showed variability. Doyle and Chatzopoulos found the success rate of NSRCT was lower than STI (73% vs. 98%) [25, 26]. While studies conducted by Hanahan and Vahdati showed the two treatment modalities exhibited a comparable result 99.3% vs. 98.4% [24, 27]. Clearly costs vary significantly in different countries and effectiveness data changed in different study. In a previous study conducted in 2009, Pennington built a Markov model to simulate the lifetime path of teeth following different treatment decisions based on the healthcare system operating in the UK. The cost was based on the UK National Health Service (NHS) and the effectiveness is longevity of the tooth. The model showed that root canal treatment is an appropriate and cost-effective intervention to extend the life of a maxillary incisor tooth with a diseased pulp. It extended the life of the tooth at an additional cost of £5–8 per year of tooth life. At current costs the role of implant is limited to a third line intervention if re-treatment fails [14]. In this Markov model. Treatment outcomes were taken from systematic reviews and typical staff time and resource costs were estimated based on UK NHS secondary healthcare setting. While in the present study, a clinical cohort study was conducted to obtain the data for cost-effectiveness analysis. Initial treatment costs and maintenance costs, including medical and time costs were calculated over a 5-year period were used as measure of cost.

This research was based on the 5-year cost and outcome data of hospital in Beijing, China. The sample size of this study was small, which may affect the results of the study. The research data may be more abundant and of great significance for patients with longer follow-up time. The costs incorporated within the study were specific to the healthcare system currently operating in China and the study was conducted under the present circumstance. Changes of medical policies may lead to different results, such as changes in medical prices. Therefore, this result should be interpreted with caution in different times and economic, medical backgrounds.

## Conclusions

In conclusion, NSRCT was more cost-effective than STI for managing pulp and periapical diseases considering patient’s willingness to pay.

## Data Availability

The datasets used and/or analysed during the current study are available from the corresponding author on reasonable request.

## List of abbreviations

NSRCT: Nonsurgical root canal treatment
STI: Single-tooth implant
MBL: Marginal bone loss
CNY: China Yuan
ICER: Incremental cost effectiveness ratios
WTP: Willingness to pay
PSM: Propensity Score Matching
